# T1/T2 Proportional Magnetic Resonance Nanoprobe Monitoring Tumor Autophagy during Chemotherapy

**DOI:** 10.3390/nano14201673

**Published:** 2024-10-18

**Authors:** Jia Cui, Taixing Zhang, Fei Wang, Lingzi Feng, Guangjun Deng, Ting Wu, Le Yin, Yong Hu

**Affiliations:** 1 MOE Key Laboratory of High Performance Polymer Materials and Technology, College of Engineering and Applied Sciences, Nanjing University, Nanjing 210023, China; dg1934018@smail.nju.edu.cn (J.C.); zhangtaixing0513@163.com (T.Z.); 602022340071@smail.nju.edu.cn (F.W.); flzflzii@outlook.com (L.F.); 2 Tongzhou Renming Hospital, 999 Jianshe Road, Jinsha Town, Tongzhou District, Nantong 226030, China; yummygjy@sina.com; 3 Department of Radiology, Affiliated Hospital of Nanjing University of Chinese Medicine, Nanjing 210068, China; 4 Nanjing University (Suzhou) High-Tech Institute, Renai Road 150, Suzhou Industrial Park, Suzhou 215123, China

**Keywords:** nanoparticles, magnetic resonance imaging, autophagy, nitroxide radicals, reactive oxygen species

## Abstract

Autophagy leads to cellular tolerance of the therapeutic pressure of chemotherapeutic drugs, resulting in treatment resistance. Therefore, the effective monitoring of the autophagy status of tumors in vivo and the regulating of the autophagy level are crucial for improving the efficacy of chemotherapy. In this work, we grafted nitroxide radicals onto the surface of iron oxide nanoparticles (Fe_3_O_4_ NPs) using dendrimer polymers, yielding Fe_3_O_4_-NO· NPs that are responsive to reactive oxygen species (ROS) and possess enhanced T1 and T2 signal capabilities in a magnetic resonance imaging (MRI) measurement. The ROS in tumor cells generated by autophagy quenches the nitroxide radicals, thereby weakening the T1 signal. In contrast, Fe_3_O_4_ NPs are unaffected by intracellular ROS, leading to a stable T2 signal. By comparing the intensity ratio of T1 to T2 in Fe_3_O_4_-NO· NPs, we can evaluate the in vivo autophagy status within tumors in real time. It also revealed that Fe_3_O_4_-NO· NPs loaded with doxorubicin (Dox) and combining the autophagy inhibitor exhibited high antitumor activity in cells and tumor-bearing mice. This system, which combines real-time monitoring of tumor cell autophagy with the delivery of chemotherapeutic drugs, provides an innovative and effective strategy for tumor treatment with potential clinical application prospects.

## 1. Introduction

Autophagy is a process in which cells degrade some of their components through autolysosomes, serving as a crucial means to maintain normal cellular physiological functions [1]. It inhibits the accumulation of early mutations in normal cells and the proliferation of early state cancer cells, thereby inducing apoptosis [2]. However, in mature tumor tissues, cancer cells utilize autophagy to degrade and recycle intracellular substrates and damaged organelles, mitigating the cellular stress caused by nutrient deprivation, hypoxia, radiation, and cytotoxic factors, which helps them avoid cell death and induces drug resistance [3,4]. Therefore, autophagy acts as an important mechanism for the therapeutic tolerance and evasion of apoptosis in the established tumor, promoting tumor progression [5,6,7]. Consequently, the autophagic pathway in tumor cells has emerged as a key target for cancer therapy and the real-time monitoring of tumor autophagic activity and modulating its characteristics during cancer treatment is crucial for enhancing therapeutic efficacy [8,9].

Currently, several methods have been developed for detecting autophagy, including the Western Blot analysis of autophagy-related LC3 proteins [10,11], the immunofluorescence tracking of GFP-tagged LC3 proteins [12], and the observation of autolysosomes under transmission electron microscopy [13]. While these methods provide accurate information on cellular autophagy, they struggle to monitor real-time changes in autophagy levels in vivo. Thus, developing a method to dynamically observe intracellular autophagy levels in tumor cells poses significant clinical challenges. Recent research has suggested a positive correlation between intracellular ROS levels and autophagic intensity [14,15,16]. Elevated ROS levels stimulate autophagy activation, increasing autophagic vesicles that form autolysosomes to maintain cellular homeostasis. Thus, monitoring changes in intracellular ROS levels can effectively reflect alterations in autophagic intensity [17]. For instance, the characteristic UV absorption peak of gold–silver core-shell nanoprobes exhibits red shifts as the silver nanoshell is oxidized and etched by ROS, allowing the semi-quantitative analysis of the autophagy levels at the cellular level [18]. Additionally, immunofluorescence techniques can directly monitor intracellular ROS levels, but these methods are still challenging to apply for in vivo ROS detection [19].

**Scheme 1:**
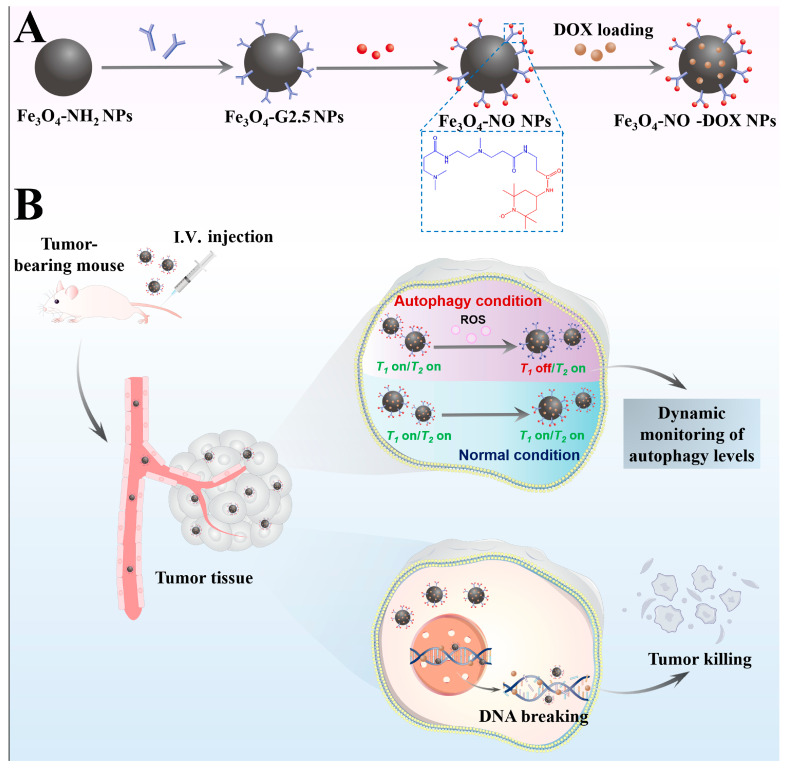
Schematic illustration of the synthesis of Fe_3_O_4_-NO·NPs (**A**) and monitoring the in vivo autophagy in tumor tissue (**B**).

Magnetic Resonance Imaging (MRI), a non-invasive diagnostic technique, enables long-term, real-time monitoring of tumor growth and is a robust clinical tool for tumor diagnosis [20,21]. Recently, nitroxide radicals have been employed as MRI contrast agents in tumor diagnosis to enhance T1 signals. These radicals become inactive upon oxidation by intracellular ROS, leading to the disappearance of the T1 signal [22,23,24]. Therefore, in this work, we developed an ROS-responsive MRI contrast agent, Fe_3_O_4_-NO· NPs, with both T1 and T2 signal enhancement capabilities, for monitoring autophagy in tumor cells. These Fe_3_O_4_-NO· NPs were synthesized by grafting nitroxide radicals onto dendrimer-coated Fe_3_O_4_ NPs (Scheme 1A). The nitroxide radicals provide T1 signals, while the Fe_3_O_4_ NPs contribute T2 signals. In tumor cells, elevated autophagy generates substantial ROS, quenching the nitroxide radicals on Fe_3_O_4_-NO· NPs and thus weakening the T1 signal. Since Fe_3_O_4_ NPs are unaffected by ROS, T2 signal intensity remains stable (Scheme 1B). To avoid false positive or negative results due to changes in probe concentration caused by the metabolism or local aggregation/diffusion of Fe_3_O_4_-NO· NPs, we analyzed the ratio of T1 to T2 signal intensities, which accurately reflects the ROS-associated autophagy variations. Furthermore, we synthesized doxorubicin-loaded Fe_3_O_4_-NO· NPs (Fe_3_O_4_-NO·-Dox) and introduced autophagy modulators during chemotherapy to investigate the relationship between tumor autophagy and chemotherapeutic efficacy. This system leverages MRI technology to detect real-time changes in autophagy during chemotherapy, revealing the connection between tumor cell autophagy and chemotherapy outcomes, offering a novel approach to optimizing cancer chemotherapy.

## 2. Experimental Procedures

### 2.1. Materials

Ferric chloride (FeCl_3_·6H_2_O), Ferrous chloride (FeCl_2_·4H_2_O), 1,6-hexanediamine, Potassium permanganate (KMnO_4_), Anhydrous sodium acetate, Methyl methacrylate, Ethylenediamine, and 4-amino-2,2,6,6-tetramethylpiperidinooxy free radical were all purchased from J&K Science (Shanghai, China). 2,7-duchlorodihydrofluorscein diacetate (H_2_DCFDA), Rapamycin, 3-methyladenine (3-MA), Doxorubicin Hydrochloride (Dox), EBSS balanced salt solution, Nacetylcysteine (NAC, 99.0%), N-(3-Dimethylaminopropyl)-N-ethylcarbodiimide hydrochloride (EDC), N-hydroxy-succinamide (NHS, 98.0%) were purchased from Sigma Aldrich (St. Louis, MO, USA).

### 2.2. Synthesis of Fe_3_O_4_-NO· NPs and Fe_3_O_4_-NO -Dox NPs

Firstly, amino group-modified Fe_3_O_4_ NPs were synthesized according to the literature [20]. Briefly, 1,6-hexanediamine (6.5 g), anhydrous sodium acetate (2.0 g) and FeCl_3_·6H_2_O (1.0 g) were dissolved in glycol (30 mL) and stirred vigorously at 50 °C to obtain a transparent solution. This solution was then transferred into a Teflonlined autoclave and reacted at 200 °C for 6 h. The obtained magnetite nanoparticles were then rinsed with water and ethanol (3 times) repeatedly to effectively remove the solvent and the unbound 1,6-hexanediamine, and then dried at 50 °C before use. During each rinsing step, the nanoparticles were magnetically separated from the supernatant. The obtained samples were named Fe_3_O_4_-NH_2_ NPs.

0.5 g Fe_3_O_4_-NH_2_ NPs was dispersed in 10 mL of absolute ethanol with 1 mL of methyl acrylate in methanol (20%, *v*/*v*). This mixture was sonicated for 1 h, and then mechanically stirred for 48 h. The obtained sample was magnetically separated and washed with methanol 3 times to obtain the 0.5th generation dendrimer modified Fe_3_O_4_ nanoparticles. After that, this sample was dispersed in 10 mL of methanol and 2 mL of ethylenediamine (50%, *v*/*v*, in methanol) was added to this dispersion. This mixture continued to stir for 3 h. Finally, the sample was magnetically separated and washed with methanol 3 times to obtain the dendrimer-modified Fe_3_O_4_ nanoparticles (G1). The above steps were repeated to get the 2.5th generation dendrimer modified Fe_3_O_4_ NPs (Fe_3_O_4_-G2.5 NPs).

The Fe_3_O_4_-G2.5 NPs were hydrolyzed in a 1% potassium permanganate aqueous solution for 24 h, magnetically separated, and washed with deionized water 3 times. Then this sample was dispersed in 30 mL of DI water with 100 mg of 1-(3-dimethylaminopropyl)-3-ethylcarbodiimide hydrochloride (EDC), 60 mg of N-hydroxysuccinimide (NHS) and 3 mL (10 mg/mL) of 4-amino-2,2,6,6-tetramethylpiperidinyloxy radical. After 6 h of mechanical stirring at room temperature, the samples were magnetically separated and washed with phosphate buffered saline (PBS) several times to remove excess reactants. Finally, nitroxide radical-modified magnetic nanoparticles (abbreviated as Fe_3_O_4_-NO· NPs) were obtained.

**To synthesize** Fe_3_O_4_-NO·-Dox NPs, 10 mg of Fe_3_O_4_-NO· NPs was dispersed in 10 mL of PBS solution (pH 7.4), and 2 mg of Dox was added into this suspension with continuous stirring at room temperature. After 6 h, this suspension was separated by centrifugation at 10,000× *g*. The supernatant was collected and the Dox concentration in the supernatant was calculated according to the standard curves of Dox obtained by the UV absorption measurement (JASCO V-670). The Dox loading content and loading efficiency were obtained by the following equations.

$$\begin{array}{c} Loading\;content=\frac{weight\;of\;Dox\;in\;the\;{\mathrm{Fe}}_{3}O_{4}-NO\cdot -DoxNPs}{weight\;of\;\;{\mathrm{Fe}}_{3}O_{4}-NO\cdot -DoxNPs}*100\\ Loading\;efficiency=\frac{weight\;of\;Dox\;in\;the\;{\mathrm{Fe}}_{3}O_{4}-NO\cdot -DoxNPs}{Total\;weight\;of\;Dox\;added}*100 \end{array}$$

The loading efficiency of Dox was 57.5% and the loading content of Dox was 11.2%.

### 2.3. Characterization

**The morphology** of these as-synthesized Fe_3_O_4_-NO· NPs were investigated by transmission electron microscopy (TEM; JEOL TEM-100). The size distribution was determined by a dynamic light scattering (DLS) method (NanoZS90; Malvern). The X-ray diffraction (XRD) pattern was recorded among the 2θ ranging from 20 to 80 degrees on a Hitachi X-ray diffractometer at 40 kV and 200 mA. Fourier transform infrared spectroscopy (FT-IR) was performed through mixing the dry powder of the samples with KBr on a NEXUS-870 Fourier Transform Infrared Spectrometer. The magnetic measurement was carried out at room temperature by a vibrating sample magnetometer (VSM; Lakeshore 7400, Columbus, OH, USA). The optical properties of the Fe_3_O_4_-NO· NPs were measured by a UV-vis-NIR spectrophotometer (JASCO V-670). The signal of NO· in the Fe_3_O_4-_NO· NPs was tested using electron paramagnetic resonance (EPR) and the spectrum was recorded at room temperature by using a Bruker EMXplus-10/12 spectrometer (Bruker; Germany) at 9.85 GHz with a variable temperature control unit (Bruker ER4141 VT-I, Germany).

### 2.4. Animal Models

To set up the 4T1 tumor-bearing mice model, female BALB/c mice (4–6 weeks old) were purchased from the Comparative Medicine Centre of Yangzhou University, and raised in a specific pathogen-free (SPF) facility. Firstly, 4T1 mouse breast cancer cells were subcutaneously inoculated into the right armpit of the mice (100 μL, 1 × 10^7^ cells per mouse). When the tumor volumes reached about 60–100 mm^3^, these mice were randomly divided into different groups to perform the in vivo experiments. All the animal experiments were reviewed and approved by the Committee on Animals at Nanjing University (IACUC-D2103066) and the guidance and support were provided by the National Institute of Animal Care.

### 2.5. Identification of the In Vitro Autophagy

Ad-mCherry-GFP-LC3B Transfection was performed to monitor the generation and degree of autophagy in the 4T1 cells after the incubation by EBSS. For Ad-mCherry-GFP-LC3II transfection, 4T1 cells were grown in 6-well plates and reached 20–30% confluence for transfection. The cells were transfected with AdmCherry-GFP-LC3-II adenovirus (Beyotime, Shanghai, China) at a MOI of 20 for 12 h at 37 °C. After the incubation by EBSS, autophagy was observed with fluorescence microscope (DMi8 Leica, Germany). Autophagy flux was evaluated by calculating the number of green and red points.

### 2.6. In Vitro Cytotoxicity Assay

The cell proliferation–toxicity test kit (CCK-8) is used to determine the toxicity of Fe_3_O_4_-NO·NPs against normal cells and tumor cells. First, normal human tissue hepatocytes (L02) and 4T1 cells were seeded into a 96-well plate at a density of 1 × 10^4^ cells per well, and cultured at 37 °C, 5% CO_2_ for 24 h. Then, the cells were washed by PBS 3 times, and subsequently incubated with different concentrations of Fe_3_O_4_-NO· NPs and Fe_3_O_4_-NH_2_ NPs solutions at 37 °C and 5% CO_2_ for 24 h. After that, the cells were further washed by PBS 3 times. Finally, 100 μL of 10% CCK-8 medium solution was added to each well and the cells were incubated for 1 h. The absorbance of each well was measured with a microplate reader using a dual-wavelength method. The detection wavelength was 450 nm and the reference wavelength was 620 nm. The result was the average of the three measurement results.

### 2.7. In Vitro MRI Study

For normal 4T1 cells and 4T1 cells treated with EBSS starvation for 4 h, they were treated with PBS and NAC, respectively, and used for MRI scanning on a 9.5 T MR scanner (94/20 USR, Bruker Biospec, Germany). In the turbine spin echo sequence, the parameters are as follows: field of view = 32 mm × 32 mm; repetition time = 2600 ms; echo time = 33 ms; echoes = 11 ms; number of averages = 3; slice thickness = 0.7 mm. The slice gap is the default value. For T2-weighted MR imaging, the scan duration is 43 s. In the turbo spin echo sequence, the parameters are as follows: field of view = 32 mm × 32 mm; repetition time = 1793 ms; echoe time = 17.2 ms; echo gap = 8.6 ms; number of averages = 3; slice thickness = 0.7 mm. The slice gap is the default value. For T1-weighted MR imaging, the scan duration is 49 s.

### 2.8. In Vivo MRI Study

The MR scanner at 9.5 T is used for in vivo MR scanning. The parameters for the T2-weighted MR imaging turbo spin echo sequence are as follows: field of view = 32 mm × 32 mm; repetition time = 2600 ms; echo time = 33 ms; echo gap = 11 ms; number of averages = 3; slice thickness = 0.7 mm; the slice number is 24; matrix size = 180 × 180; the scanning duration is 2 min and 51 s. The parameters for the T1-weighted MR imaging turbo spin echo sequence are as follows: field of view = 32 mm × 32 mm; repetition time = 1793 ms; echo time = 17.2 ms; echo gap = 8.6 ms; number of averages = 3; slice thickness = 0.7 mm; the slice number is 24; matrix size = 300 × 320; the scanning duration is 4 min and 45 s.

### 2.9. In Vitro ROS Detection

The ROS level was verified via a reactive oxygen species assay kit (Beyotime Biotechnology, Shanghai, China) according to the manufacturer’s instructions. The intracellular generation of ROS in 4T1 cells incubated with Fe_3_O_4_-NO· NPs and NAC under either a normal condition or an autophagy condition was assessed using H_2_DCFDA as a probe. Briefly, 4T1 cells were seeded in a 6-well plate with a density of 5 × 10^4^ cells per well. After 24 h of incubation, half of the 4T1 cells were subjected to EBSS for another 6 h to induce autophagy. After 4 h, the cells were further incubated with 10 μL of H_2_DCFDA (10 mM in DMSO) for 1 h at 37 °C, followed by being washed by PBS twice. The ROS was immediately measured by confocal laser scanning microscopy (CLSM, em/ex, 488 nm/520 nm, LSM 700, Leica).

### 2.10. In Vitro Antitumor Effect

A cell proliferation–toxicity test kit (CCK-8) was used to determine the toxicity of 3-MA and rapamycin against 4T1 cells. First, mouse breast cancer cells were seeded in a 96-well plate at a density of 1 × 10^4^ cells/well, and cultured at 37 °C and 5% CO_2_ for 24 h. Then, the cells were cultured for 24 h in a medium containing different concentrations of 3-MA or rapamycin. After washing the cells 3 times with PBS, we added 100 μL of 10% CCK-8 medium to each well and incubated them for 1 h. Then, a dual-wavelength microplate reader was used to measure the absorbance. The detection wavelength is 450 nm and the reference wavelength is 620 nm. The toxicity of Dox and Fe_3_O_4_-NO·-Dox combined with 3-MA or rapamycin on 4T1 cells was also measured.

### 2.11. In Vivo Antitumor Effect

Suspensions of 4T1 tumor cells were subcutaneously inoculated into the right armpit of the mice (100 mL, 1 × 10^7^ cells per mouse). When the tumor sizes reached a mean volume of ~100 mm^3^, all the mice were randomly divided into four groups containing five mice in each group: 1. control group (0.1 mL/mouse/time, PBS); 2. Fe_3_O_4_-NO·-DOX group (0.1 mL/mouse/time, containing 10 μg/mL Dox); 3. Fe_3_O_4_-NO·-DOX +Rapa group (0.1 mL/mouse/time, containing 10 μg/mL Dox and 200 nM Rapamycin); 4. Fe_3_O_4_-NO·-DOX + 3-MA group (0.1 mL/mouse/time, containing 10 μg/mL Dox and 1 mM 3-MA). Samples were intratumorally injected into the mice every three days and this procedure was repeated three times. The body weights, tumor volumes, and survival rates were recorded. After 15 days, for histological analyses, the mice were sacrificed, and their tumors and main organs were collected, washed three times with PBS and weighed and fixed in 10% neutral buffered formalin solution. The H&E staining assays were obtained by slicing the tissues. Images of H&E were obtained via fluorescence microscopy (DMi8, Leica).

### 2.12. Statistical Analysis

The data were expressed as mean ± SD. The statistical calculation of experimental data used one-way ANOVA statistical analysis. The data were classified with *p* values and denoted by (*) for *p* < 0.05, (**) for *p* < 0.01, and (***) for *p* < 0.001.

## 3. Results and Discussion

**Figure 1:**
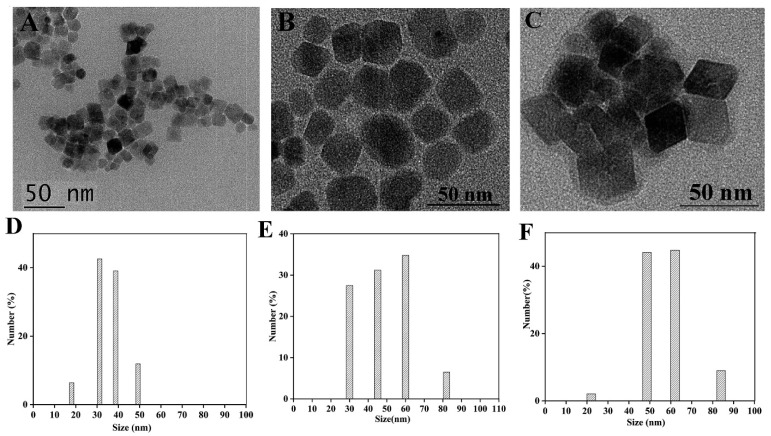
TEM images of Fe_3_O_4_ NPs (**A**), Fe_3_O_4_-NH_2_ NPs (**B**) and Fe_3_O_4_-NO· NPs (**C**). The size distributions of Fe_3_O_4_ NPs (**D**), Fe_3_O_4_-NH_2_ NPs (**E**) and Fe_3_O_4_-NO· NPs (**F**) determined by DLS. All results were mean values of three measurements.

The Fe_3_O_4_ NPs were prepared using the solvothermal method and they were modified with amino groups to form Fe3O4-NH2 NPs, which were used to graft dendritic polymers onto their surface. Finally, nitroxide radicals were connected to these dendrimer-modified Fe_3_O_4_-NH_2_ NPs to obtain their multifunctional nano-theranostic agent (Fe_3_O_4_-NO· NPs). These pristine Fe_3_O_4_ NPs have regular cubic structures and uniform particle sizes of approximately 20 nm, as characterized by TEM (Figure 1A). The dynamic light scattering result indicates a mean particle size of about 37 nm (Figure 1D). After the modification of the amino group, the shape and size of the Fe_3_O_4_-NH_2_ NPs remain consistent with the original Fe_3_O_4_ NPs as seen in the TEM image (Figure 1B). However, the hydrated diameter from DLS results increased significantly to about 52 nm, indicating the successful modification of the amino groups on the surface of the Fe_3_O_4_ NPs (Figure 1E). Compared to Fe_3_O_4_-NH_2_ NPs, the morphology and size of the Fe_3_O_4_-NO· NPs do not show obvious changes (Figure 1C), but their hydrated radius increases slightly to around 57 nm (Figure 1F). After placing these Fe_3_O_4_-NO· NPs in aqueous suspension at room temperature for a week, we found no significant changes in their morphology and size, indicating that these Fe_3_O_4_-NO· NPs possessed good stability.

**Figure 2:**
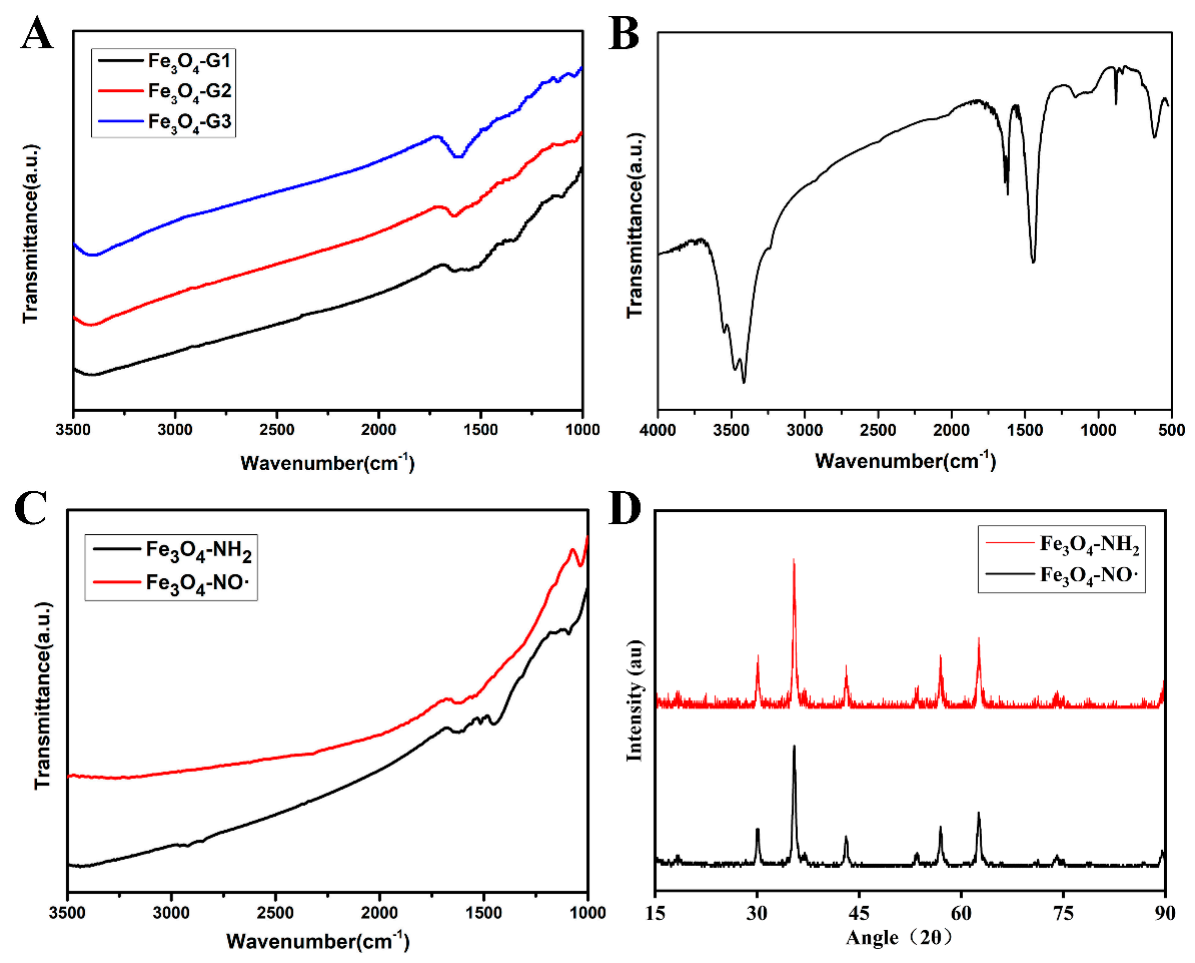
IR spectra of the Fe_3_O_4_-G1, Fe_3_O_4_-G2, Fe_3_O_4_-G3 NPs (**A**); PAMAM obtained after the hydrochloric acid dissolution of Fe_3_O_4_-NH_2_ NPs (**B**) and Fe_3_O_4_-NH_2_, Fe_3_O_4_-NO· NPs (**C**). XRD patterns of Fe_3_O_4_-NH_2_ and Fe_3_O_4_-NO· NPs (**D**).

Infrared spectroscopy was applied to study Fe_3_O_4_ NPs grafted with different generations of polyamidoamine (PAMAM). As can be seen from Figure 2A, the absorption intensity at ~1620 cm^−1^ representing the characteristic peak of the amide bond from PAMAM became increasingly prominent in the infrared spectrum as the generation of PAMAM increased. To eliminate the interference of Fe_3_O_4_ NPs and more directly demonstrate the presence of PAMAM on the surface of Fe_3_O_4_, we dissolved 2.5 generations of PAMAM-modified Fe_3_O_4_-NH_2_ NPs in hydrochloric acid and conducted FT-IR characterization of the resulting pure 2.5-generation PAMAM (Figure 2B). The peak at ~620 cm^−1^ representing the presence of tertiary amides, the peak at ~1445 cm^−1^ representing the presence of carboxylic acids after acidolysis, the peak at ~1640 cm^−1^ attributing to the presence of amide bonds, and the peak at ~3400 cm^−1^ relating to the asymmetric stretching vibration of N-H in PAMAM are all clearly observed. These results collectively confirm that the dendritic polymer PAMAM had been successfully grafted onto the surface of Fe_3_O_4_ NPs.

Figure 2C depicts the FTIR spectra of Fe_3_O_4_-NH_2_ NPs and Fe_3_O_4_-NO· NPs. The two peaks at 1456 cm^−1^ and 1622 cm^−1^ in the Fe_3_O_4_-NH_2_ NPs curve confirm the presence of -NH_2_ on the surface of the Fe_3_O_4_ NPs, while the peak at 1633 cm^−1^ in the Fe_3_O_4_-NO· NPs’ curve verifies the successful modification of NO·. We also characterized the crystal structures of Fe_3_O_4_-NH_2_ NPs and Fe_3_O_4_-NO· NPs by XRD (Figure 2D) and found that both the NPs have a spinel inverse structure of ferric oxide, suggesting that the crystal type of these samples did not change after the grafting and modification procedures.

**Figure 3:**
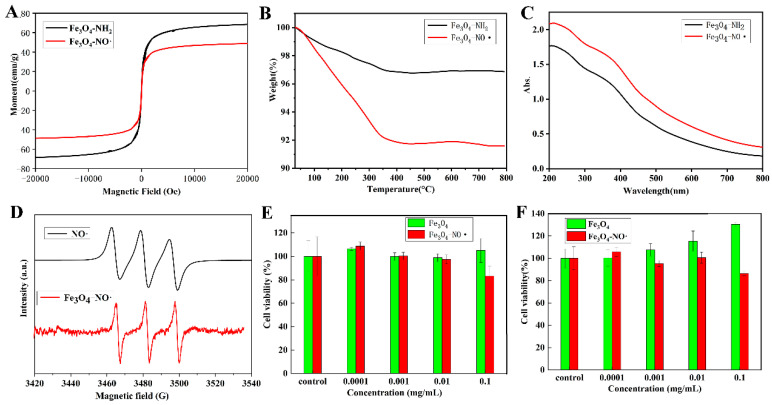
(**A**) The magnetic hysteresis loops of Fe_3_O_4_-NH_2_ and Fe_3_O_4_-NO· NPs. (**B**) UV–vis absorption spectra of Fe_3_O_4_-NH_2_ and Fe_3_O_4_-NO· NPs. (**C**) TG curves of Fe_3_O_4_-NH_2_ and Fe_3_O_4_-NO· NPs. (**D**) EPR spectra of NO· and Fe_3_O_4_-NO· NPs. (**E**) In vitro viability of L02 cells incubated with Fe_3_O_4_-NH_2_ and Fe_3_O_4_-NO· NPs. (**F**) In vitro viability of 4T1 cells incubated with Fe_3_O_4_-NH_2_ and Fe_3_O_4_-NO· NPs.

Because ultrafine Fe_3_O_4_ NPs have unique superparamagnetic properties, we measured the magnetic properties of these samples at room temperature using a vibrating sample magnetometer (VSM) (Figure 3A). According to the TG curves of Fe_3_O_4_-NH_2_ and Fe_3_O_4_-NO· NPs (Figure 3B), the weight ration of Fe_3_O_4_ in Fe_3_O_4_-NH_2_ NPs is about 97%, while the Fe_3_O_4_ in Fe_3_O_4_-NO· NPs is about 92%. The saturation magnetization of Fe_3_O_4_ in Fe_3_O_4_-NH_2_ NPs is 70.1 emu/g at 25 °C, while the saturation magnetization of Fe_3_O_4_ in Fe_3_O_4_-NO· NPs is approximately 54.3 emu/g at 25 °C. We believe that the decrease in the saturation magnetization of Fe_3_O_4_ NPs may be attributed to the process of grafting nitroxide radicals onto Fe_3_O_4_-NH_2_ NPs. It can be also observed from the results that both the samples exhibit zero coercivity, confirming their superparamagnetic behaviors. The saturation magnetization of Fe_3_O_4_-NO· NPs is smaller than that of Fe_3_O_4_-NH_2_ NPs. We speculate that this may be due to the decreased effective magnetic content in Fe_3_O_4_-NO· NPs resulting from the grafting and modification of dendritic polymers and nitroxide radicals, which leads to a reduction in magnetization. To verify this hypothesis, we conducted thermogravimetric analysis (TGA) tests on Fe_3_O_4_-NH_2_ NPs and Fe_3_O_4_-NO· NPs (Figure 3B). Obviously, Fe_3_O_4_-NO· NPs exhibited about 5% more weight loss compared to Fe_3_O_4_-NH_2_ NPs. The TGA results confirm that the effective magnetic component in Fe_3_O_4_-NO· NPs is smaller than that in Fe_3_O_4_-NH_2_ NPs, which is partly responsible for the lower saturation magnetization. Figure 3C shows the UV-vis absorption curves of Fe_3_O_4_-NH_2_ NPs and Fe_3_O_4_-NO· NPs. It can be seen that the Fe_3_O_4_-NH_2_ NPs and Fe_3_O_4_-NO· NPs do not exhibit significant absorption in the wavelength range of 220–800 nm, indicating that there are no unsaturated groups or other complex structures formed on the surface of Fe_3_O_4_ NPs throughout the entire reaction process. The indistinct shoulder peak appearing at 300 nm may be due to the ion pairs formed by the dendrimer polymer and metal particles. The presence of nitric oxide radicals in Fe_3_O_4_-NO· NPs can be verified by electron paramagnetic resonance (EPR) measurement. As can be seen from the spectrum (Figure 3D), both the samples exhibit the characteristic triplet curve of NO·, confirming that NO· has been successfully modified onto the PAMAM-grafted Fe_3_O_4_ NPs.

Before these Fe_3_O_4_-NO· NPs could be used for in vivo studies, we employed a cell proliferation–toxicity detection test to evaluate the cytotoxicity of the Fe_3_O_4_-NH_2_ NPs and the Fe_3_O_4_-NO· NPs. As can be seen from the test, unmodified Fe_3_O_4_ NPs exhibit no cytotoxicity to normal cells (L02, Figure 3E) and tumor cells (4T1, Figure 3F), and even promote the growth of normal cells. We speculate that the iron element may be beneficial for cell growth. The cell viability of L02 and 4T1 the cells treated with Fe_3_O_4_-NO· NPs at concentrations below 0.1 mg/mL was higher than 80%, further confirming the low cytotoxicity of Fe_3_O_4_-NO· NPs.

**Figure 4:**
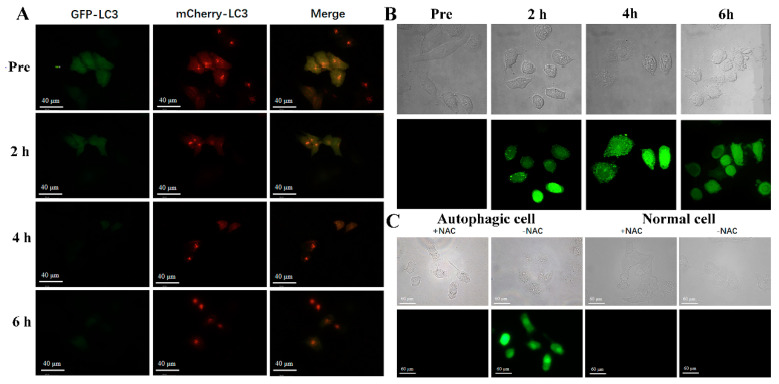
(**A**) Images of ad-mCherry-GFP-LC3-II-transfected 4T1 cells upon the induction of autophagy with EBSS for different time intervals. (**B**) CLSM images of ROS in 4T1 cells upon the induction of autophagy with EBSS for different time intervals. (**C**) CLSM images of ROS in 4T1 cells cultured with (+NAC) or without (−NAC) NAC under normal or autophagy conditions, respectively. Green: ROS indicator H_2_DCFDA in (**B,C**).

To verify the correlations between cell autophagy and intracellular ROS levels, we cultured 4T1 cells with EBSS for varying duration time to induce autophagy. After that, the lysosomes of the cells were transfected with Ad-mCherry-GFP-LC3B adenovirus. By observing the color changes of the lysosomes, we monitored the progression of the autophagy. The acidic environment in the lysosome would cause the green fluorescence protein (GFP) to quench while the fluorescence produced by mCherry (red) remained unaffected by acidity. As the autophagy progressed, the autophagosomes bonded to the lysosomes, leading to the increased acidity of the lysosomes and the continuous reduction in green fluorescence intensity. As shown in Figure 4A, the longer time the HepG2 cells were cultured under EBSS starvation, the weaker the green fluorescence in the cells became, while the intensity of red fluorescence remained relatively stable. This indicates that the EBSS method effectively induced autophagy in the cells, and robust autophagy occurred after 4 h of culture.

Furthermore, H2DCFDA was used as a probe to qualitatively analyze the production of ROS induced by autophagy in cells (Figure 4B). We found that as autophagy was induced in cells by EBSS, the green fluorescence of the cells increased, indicating an increase in the intracellular ROS levels, and the fluorescence intensity was the highest at 4 h. Combining the results of Figure 4A, we can conclude that as tumor autophagy levels become strong, the ROS levels of the tumor cells increased. To further verify whether the ROS levels inside tumor cells are directly affected by autophagy, we treated normally cultured 4T1 cells and EBSS starvation-treated 4T1 cells with an antioxidant (NAC). The intracellular ROS production was determined by a H2DCFDA fluorescent probe (Figure 4C). We found that in normally cultured 4T1 cells, regardless of NAC treatment, no significant ROS signals were detected in the cells. However, in autophagic 4T1 cells without NAC co-culture, we detected a significant ROS signal (green fluorescence), while autophagic 4T1 cells co-cultured with NAC did not exhibit green fluorescence. The above experimental results suggest that the ROS levels inside tumor cells are directly related to the degree of autophagy, and we can indirectly detect tumor cell autophagy by measuring the ROS content inside tumor cells, which support the use of Fe_3_O_4_-NO· NPs to detect autophagy in tumor cells.

**Figure 5:**
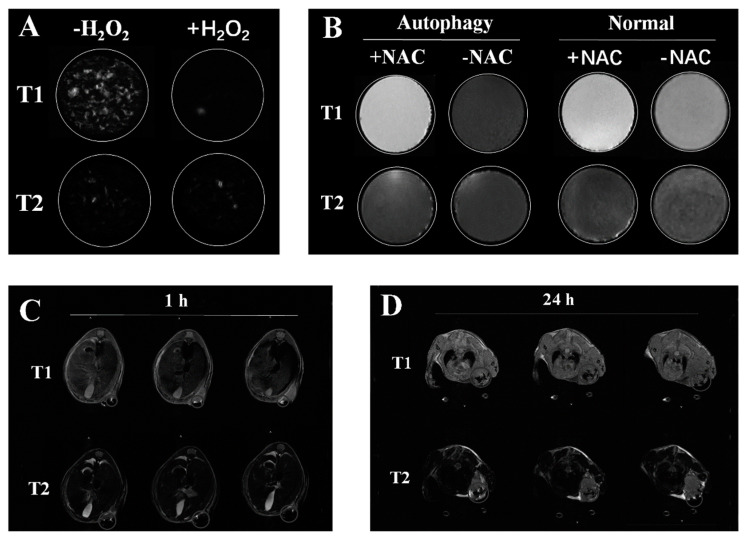
(**A**) T1 and T2 weighted mapping of Fe_3_O_4_-NO· NPs before and after H_2_O_2_ oxidation. (**B**) T1 and T2 weighted mapping of 4T1 cells co-cultured with Fe_3_O_4_-NO· NPs in the presence (+NAC) or absence (−NAC) of NAC under normal or autophagy conditions. T1 and T2 weighted MR images of mice bearing 4T1 tumors (circle) at 1 h (**C**) and 24 h (**D**) post-administration of Fe_3_O_4_-NO· NPs.

Based on the above results, we investigate the responsiveness of Fe_3_O_4_-NO· NPs to ROS signals by MRI. Firstly, Fe_3_O_4_-NO· NPs were incubated with 5 µM H_2_O_2_ solution (a concentration similar to that of H_2_O_2_ in cells) for 2 h and their changes of magnetic resonance signals before and after the H_2_O_2_ treatment were measured. As shown in Figure 5A, after H_2_O_2_ treatment, the T1 signal of Fe_3_O_4_-NO· NPs became weaker, while the intensity of the T2 signal remained unaffected. This is because, after the oxidation by H_2_O_2_, the nitroxide radicals were oxidized by ROS into inert substances, leading to a weakening of the T1 signal. Meanwhile, the Fe_3_O_4_ core, which generates the T2 signal, was unaffected by ROS and maintained its initial T2 signal intensity. Therefore, we can reveal the changes in ROS content in the system by measuring the variation in the T1/T2 ratio.

Furthermore, we used the EBSS (Earle’s balanced salt solution) starvation method to induce autophagy into the 4T1 cells. Normally cultured 4T1 cells and 4T1 cells with EBSS starvation treatment were incubated with NAC, respectively, and the changes in the T1 and T2 signals of these cells were analyzed using magnetic resonance imaging (Figure 5B). After the treatment with the antioxidant NAC, the T1 signal of the 4T1 cells cultured in normal medium increased slightly compared to the T1 signal before treatment. This is because tumor cells are inherently in a state of oxidative imbalance, with relatively high ROS concentrations. ROS consumes the nitroxide radicals of Fe_3_O_4_-NO· NPs, leading to a decrease in the T1 signal. The addition of NAC depletes ROS in the cells, allowing the T1 signal of Fe_3_O_4_-NO· NPs to be maintained at its original strength. Fe_3_O_4_ NPs are insensitive to H_2_O_2_, so the T2 signal does not change significantly, regardless of NAC addition. For starved tumor cells, autophagy is enhanced, and a large amount of ROS accumulates in the cells, which consumes the nitroxide radicals in Fe_3_O_4_-NO· NPs, leading to a decrease in the T1 signal. When NAC is added, it depletes the ROS in the cells, allowing the T1 signal of Fe_3_O_4_-NO· nanoparticles to be maintained. Due to the differences in the ability of cells to uptake Fe_3_O_4_-NO· NPs and the metabolic rate of Fe_3_O_4_-NO· NPs within cells, the content of Fe_3_O_4_-NO· NPs within cells fluctuates, which also affects the intensity of the T1 signal. Therefore, the sole changes of the T1 signal cannot accurately reflect the dynamic changes in the degree of autophagy within cells. Since NO· and Fe_3_O_4_ NPs are connected by stable covalent bonds, the effects of changes in the content of Fe_3_O_4_-NO· NPs within cells on T1 and T2 signals should be the same. Considering that the T2 signal does not change with the dynamic changes in autophagy, we used the T2 signal as an internal reference. Thus, the ratio of the T1 signal to the T2 signal (R1SNR/R2SNR) can quantitatively reflect the autophagy situation within the cells. Based on this, we calculated the R1SNR/R2SNR in Figure 5B and noticed that in the 4T1 cells with autophagy induced by starvation, compared to cells with the addition of NAC, the R1SNR/R2SNR ratio decreased by more than 4.2 times in cells without NAC addition. In 4T1 cells without the starvation-induced autophagy, the R1SNR/R2SNR ratio was not affected by NAC addition (Figure 5B). These data confirm that autophagy in cells generates ROS, which consumes the nitroxide radicals in Fe_3_O_4_-NO· NPs, thereby reducing the T1 signal.

Furthermore, we validated the in vivo response characteristics of Fe_3_O_4_-NO· NPs to autophagy in mice. We collected T1- and T2-weighted MRI images of tumor tissues in tumor-bearing mice 1 h and 24 h after the injection of Fe_3_O_4_-NO· NPs to analyze the autophagy degree. As shown in Figure 5C, 1 h after the injection of Fe_3_O_4_-NO· NPs, the T1 image of the mouse tumor became brighter, indicating that the Fe_3_O_4_-NO· NPs accumulated in tumor tissue (circle) and the nitroxide radicals in Fe_3_O_4_-NO· NPs had not been quenched by ROS in a short time. However, 24 h after the injection of Fe_3_O_4_-NO· NPs (Figure 5D), the bright spots of the T1 signal in the tumor MRI image disappeared. We therefore infer that with time, the ROS in the mouse tumor cells gradually reacts with the nitroxide radical in Fe_3_O_4_-NO· NPs, leading to the gradual disappearance of the T1 signal induced by the nitroxide radical. However, from 1 h to 24 h after the injection of Fe_3_O_4_-NO· NPs (Figure 5D), we can see that the intensity of the T2 signal in the tumor tissue (circle) in mice remains unchanged, indicating that ROS in tumor tissues does not affect the T2 signal intensity of Fe_3_O_4_-NO· NPs. These data agreed with the results of the cellular experiments. Therefore, the T1 signal of Fe_3_O_4_-NO· NPs can be used to detect ROS signals in tumor tissues, indirectly revealing the autophagy status of tumor cells. Meanwhile, the T2 signal can be used to locate the position of tumor tissues in the body. By comparing the changes in the ratio of T1 to T2 signals over time, we can eliminate the interference caused by the decrease in T1 signal due to the metabolism of Fe_3_O_4_-NO· NPs.

**Figure 6:**
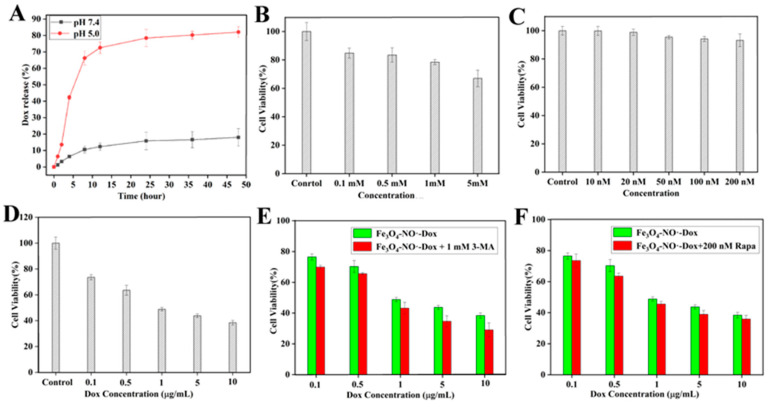
(**A**) Release of Dox from Fe_3_O_4_-NO·-Dox at different pH. In vitro viability of 4T1 cells 24 h post-incubation with 3-MA (**B**), Rapamycin (**C**) and Dox (**D**). In vitro viability of 4T1 cells 24 h post-incubation with Fe_3_O_4_-NO·-Dox plus 3-MA (**E**). In vitro viability of 4T1 cells 24 h post- incubation with Fe_3_O_4_-NO·-Dox plus Rapamycin (Rapa) (**F**).

To investigate the role of autophagy in the chemotherapy process, Dox was loaded into Fe_3_O_4_-NO· NPs to obtain Fe_3_O_4_-NO·-Dox NPs. The release of Dox from Fe_3_O_4_-NO·-Dox in PBS with different pH values was shown in Figure 6A. Compared to that in pH7.4, a faster release of Dox was observed at pH 5.0. It is reasonable that the amino groups in the Fe_3_O_4_-NO·-Dox NPs were protonated, and the dendritic polymer layer on the surface has a looser structure under acidic conditions, leading to the fast release of Dox. According to the literature reports, multidrug resistance in tumors is related to autophagy in tumor cells in chemotherapy treatment. Therefore, regulating autophagy in tumor cells holds potential clinical value in tumor therapy. In this work, rapamycin (Rapa) was applied to promote autophagy, and 3-methyladenine (3-MA) was employed to inhibit the autophagy.

Firstly, we assessed the cytotoxicity of 3-MA and Rapa to ensure that they did not significantly impact cell growth within their working concentration ranges, thereby minimizing their potential influence on therapeutic outcomes. We determined the cytotoxicity of Rapa and 3-MA as shown in Figure 6B,C. Within the tested working concentration ranges, neither 3-MA nor Rapa exhibited significant cytotoxicity against 4T1 cells, with cell survival rates remaining above 80%. Consequently, in subsequent studies, the concentration of 3-MA was maintained at 1 mM, and that of Rapa was kept at 200 nM. Then the Fe_3_O_4_-NO·-DOX NPs co-cultured with 3-MA or Rapa in the cellular experiments were conducted to evaluate the effect of autophagy on the efficacy of chemotherapy in tumor treatment.

Figure 6D illustrates the toxicity of free doxorubicin within 4T1 cells. Clearly, Dox exhibits a concentration-dependent toxicity. Over 50% of the 4T1 cells were killed when the Dox concentration exceeded 1 μg/mL. We also investigated the growth of 4T1 cells after co-incubation with Fe_3_O_4_-NO·-DOX NPs at different concentrations, combined with either 3-MA or Rapa, for 24 h, to explore the impact of autophagy on chemotherapy. As shown in Figure 6E, we observe that compared to Fe_3_O_4_-NO·-DOX NPs alone, the therapeutic effect is enhanced with increasing concentrations of Fe_3_O_4_-NO·-DOX NPs when combined with 3-MA. In contrast, the therapeutic effect of Fe_3_O_4_-NO·-DOX NPs combined with Rapa is virtually unchanged compared to Fe_3_O_4_-NO·-DOX NPs alone (Figure 6F). Therefore, we can conclude that when using Dox for anti-tumor therapy, combining it with autophagy inhibitors to suppress autophagy in tumor cells can effectively enhance its anti-tumor efficacy.

**Figure 7:**
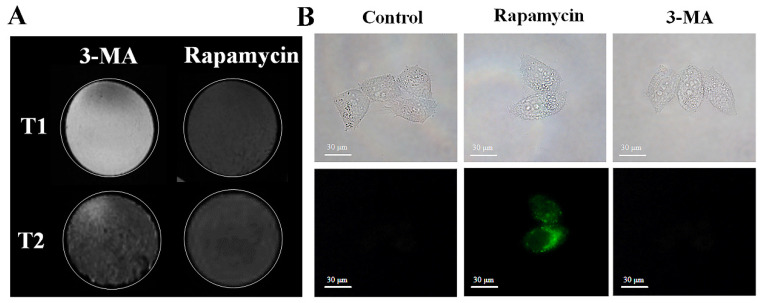
(**A**) T1 and T2 weighted MR images of 4T1 cells at 2 h post co-culture with Rapamycin or 3-MA. (**B**) Fluorescence images of 4T1 cells 2 h post the coculture with PBS, Rapamycin or 3-MA.

To verify the role of 3-MA or Rapa in regulating autophagy, we co-cultured 3-MA or Rapamycin with 4T1 cells for 2 h, then added Fe_3_O_4_-NO· NPs as contrast agents to detect the autophagy in the 4T1 cells via MRI imaging. Rapamycin inhibited the mTOR pathway of cells, thereby enhancing autophagy in the 4T1 cells. Consequently, in the MRI images (Figure 7A), the nitroxide radicals on the Fe_3_O_4_-NO· NPs in the 4T1 cells treated with Rapamycin were quenched by ROS due to increased autophagy levels, leading to weakened T1 signals. In contrast, 3-MA inhibited autophagy, reducing the level of ROS produced by autophagy in 4T1 cells. As a result, the nitroxide radicals on Fe_3_O_4_-NO· NPs remained, displaying a strong T1 signal in the MRI image.

After co-culturing 4T1 cells with 3-MA or Rapamycin for 2 h, respectively, we measured the intracellular ROS content using an H2DCFDA probe. As shown in Figure 7B, no green fluorescence was observed in the PBS group or the autophagy inhibitor 3-MA group, indicating that there was no significant ROS production in these two systems. In contrast, the 4T1 cells treated with Rapamycin for 2 h exhibited obvious green fluorescence, suggesting that the enhanced autophagy in these cells led to the generation of ROS that could react with the H2DCFDA probe. Consequently, this result is consistent with our MRI findings, demonstrating a positive correlation between the level of autophagy and the intracellular ROS content. Therefore, we can assess the degree of autophagy in cells by detecting their ROS content.

**Figure 8:**
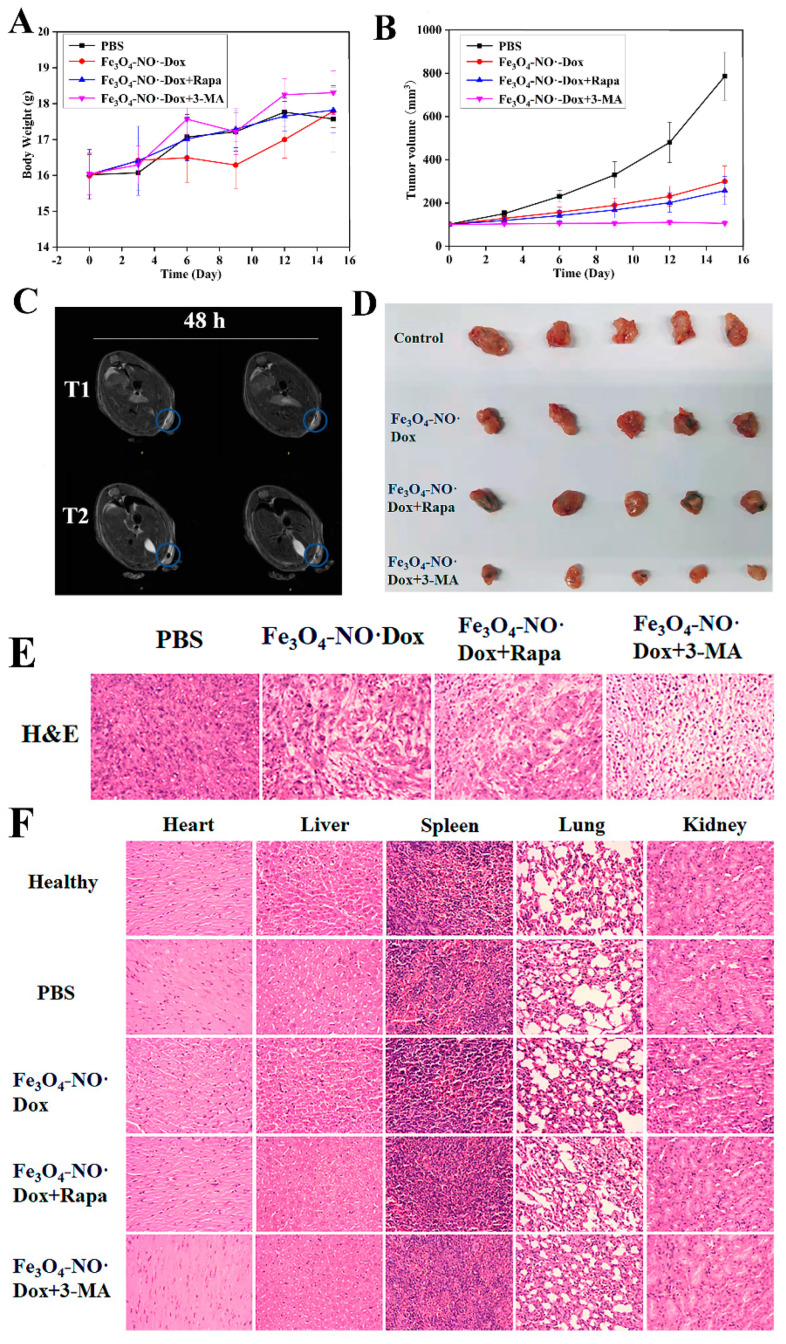
(**A**) Body weight and (**B**) tumor volume profiles of 4T1 tumor-bearing mice receiving different treatments. (**C**) MRI images of tumor tissue 48 h post the injection of Fe_3_O_4_-NO·-Dox NPs + 3-MA. (**D**) Photos of isolated tumors of 4T1 tumor-bearing mice after 15 days of different treatments. (**E**) Images of tumor tissue sections stained with H&E in different groups 15 days post-treatment. (**F**) Images of main organs (heart, liver, spleen, lung, kidney) sections stained with H&E in different groups 30 days post-treatment.

The above studies indicate that Fe_3_O_4_-NO· NPs can be utilized to detect autophagy in cells or tumor tissues in real-time. Consequently, we employed Fe_3_O_4_-NO· NPs to investigate the effects of autophagy on chemotherapy. We subjected 4T1 tumor-bearing mice to different treatments (PBS, Fe_3_O_4_-NO·-Dox NPs, Fe_3_O_4_-NO·-Dox NPs + Rapa, and Fe_3_O_4_-NO·-Dox NPs + 3-MA) to evaluate the in vivo chemotherapeutic efficacy of these therapies. After 15 days of treatment, all the mice survived without significant adverse reactions. The body weights of tumor-bearing mice in the four groups showed an overall increasing trend (Figure 8A), suggesting that none of these treatments produced overt toxic effects in mice. We also measured the tumor volumes over the treatment period and found that the tumor volume in the Fe_3_O_4_-NO·-Dox NPs + 3-MA group was significantly smaller than that in the other three groups, indicating the best therapeutic effect. In contrast, the tumor volumes in the Fe_3_O_4_-NO·-Dox NPs group and the Fe_3_O_4_-NO·-Dox NPs + Rapa group were similar and were a little smaller than that of the PBS group (Figure 8B,D). Additionally, for the Fe_3_O_4_-NO·-Dox NPs +3-MA group, we performed MRI measurements on the tumor sites 48 h after treatment. The results showed that even after 48 h of treatment, we could still observe a distinct T1 signal at the tumor site (Figure 8C, circle). In contrast to our previous experiments (Figure 5D), without 3-MA assistance, we could no longer detect the T1 signal of Fe_3_O_4_-NO· NPs in the tumor tissues after 24 h. Therefore, we believe that 3-MA effectively inhibits autophagy in tumor cells, reducing the amount of ROS generated by tumor cells, allowing Fe_3_O_4_-NO· NPs to retain their T1 signals. Furthermore, considering the anti-tumor ability of the Fe_3_O_4_-NO·-Dox NPs + 3-MA group, we suggest that combining autophagy inhibitors during tumor chemotherapy can effectively enhance the efficacy of chemotherapy.

We extracted the tumor tissues from tumor-bearing mice after 15 days of treatment with different samples, stained them with H&E, and prepared pathological sections to further evaluate the killing effects of different therapies on tumor cells. As shown in Figure 8E, the tumor tissue in the PBS group is densely packed and exhibits obvious continuity. The tumor tissues treated with Fe_3_O_4_-NO·-Dox NPs and Fe_3_O_4_-NO·-Dox NPs + Rapa exhibited significant decomposition, and some tumor cells were lysed due to cytoplasmic condensation. In the tumor tissues of mice treated with Fe_3_O_4_-NO·-Dox NPs + 3-MA, most of the tumor cells have shrunk and died, resulting in a large number of decomposed cell fragments. This result also confirmed that the Fe_3_O_4_-NO·-Dox NPs + 3-MA group exhibited the best anti-tumor therapeutic effect. After 30 days of treatment, we euthanized the mice, collected their major organs, performed tissue sectioning, and studied the pathological features of each organ using H&E staining to assess the damage to the organs caused by the different treatments. As shown in Figure 8F, the histological structures of the major organs (heart, liver, spleen, lungs, and kidneys) in the four groups of mice were not significantly different from those of the normal mice. Therefore, we concluded that none of these four treatments caused significant damage to the major organs of the mice.

The above research results indicate that inhibiting tumor autophagy during chemotherapy may enable us to reduce the dosage of chemotherapeutic drugs while maintaining their efficacy. This system provides a potential solution for enhancing the efficacy of chemotherapeutic drugs and reducing their toxic and side effects, with the potential for extension to other chemotherapeutic drug systems.

## 4. Conclusions

We have successfully synthesized multifunctional Fe_3_O_4_-NO· NPs, which are sensitive to reactive oxygen species (ROS), capable of enhancing both T1 and T2 signals, and able to load the chemotherapeutic drug doxorubicin. This system can monitor real-time changes in ROS levels within tumors through MRI, thereby revealing the autophagic status within tumor cells. In this system, the nitroxide radicals of Fe_3_O_4_-NO· NPs are readily quenched by autophagy-induced ROS, leading to a decrease in T1 signal intensity. Conversely, the T2 signal intensity of the Fe_3_O_4_ NPs remains unaffected by ROS. The ratio of T1 to T2 signal intensities reflects changes in the autophagic intensity within tumor cells. This method not only provides good spatial resolution but also offers dynamic information on the autophagic status of tumors in vivo. The combined use of the autophagy inhibitor 3-MA and Fe_3_O_4_-NO·-Dox NPs significantly reduces the therapeutic resistance of tumor cells due to autophagy, effectively enhancing the therapeutic effect of doxorubicin. However, due to the limitations in the experimental conditions, we were unable to systematically study changes in intratumoral autophagic status during doxorubicin treatment in mice using MRIs, which will be an important focus of our future research. In summary, this system effectively detects tumor autophagy in vivo, providing a novel method for studying the relationship between autophagy and a range of related diseases, as well as the impact of autophagy on chemotherapy.

## Data Availability

Data is contained within the article.
